# Treating Extensively Drug-Resistant *Acinetobacter baumannii*: Considerations for Host Characteristics and Type of Infections

**DOI:** 10.3390/pathogens15010081

**Published:** 2026-01-12

**Authors:** Anastasia Geladari, Dimitrios Kouroupis, Kyriaki Vafeidou, Vasileios Liakos, Maria Magoudi, Anastasia-Izampella Papathanasiou, Elias Iosifidis, Emmanuel Roilides, Charalampos Antachopoulos, Athina Pyrpasopoulou

**Affiliations:** 1 Infectious Diseases Unit, Hippokration General Hospital, 54642 Thessaloniki, Greece; anastasia.geladari@gmail.com (A.G.); liakospediatrics@gmail.com (V.L.); anizelidi@yahoo.gr (A.-I.P.); iosifidish@gmail.com (E.I.); roilides@gmail.com (E.R.); antachop@med.auth.gr (C.A.); 2 3rd Pediatric Department, Aristotle University, 54642 Thessaloniki, Greece; 3 2nd Propedeutic Department of Internal Medicine, Aristotle University, Hippokration General Hospital, 54642 Thessaloniki, Greece; dimcour841@gmail.com (D.K.); kiriakivfd@gmail.com (K.V.); mmagoudi@gmail.com (M.M.)

**Keywords:** *Acinetobacter baumanii*, carbapenem-resistant, treatment, synergy

## Abstract

*Acinetobacter baumannii* has been characterized by CDC, WHO and most National Healthcare Systems worldwide as a critical nosocomial pathogen, and classified as an ESKAPE (*Enterococcus faecium*, *Staphylococcus aureus*, *Klebsiella pneumoniae*, *Acinetobacter baumannii*, *Pseudomonas aeruginosa*, *Enterobacter* spp.) pathogen. Mortality of invasive infections due to *A. baumannii* exceeds 40%. To highlight its impact on public health, ECDC has organized a special project on national lab co-ordination to accurately detect and report carbapenem-resistant strains, to identify epidemiological factors for infection (or colonization) with carbapenem-resistant *A. baumanii* at clonal and sub-genomic level. This review aims to describe the history, epidemiology, and evolution of resistance of *A. baumannii*, and stress the caveats associated with the management of systemic infections. Available active antimicrobials and drugs in the pipeline are listed, and available clinical evidence on their pharmacokinetics and efficacy in various types of infections are described. Clinician’s choice of treatment (drug, and monotherapy vs. combination treatment) depends on the patients’ profile, site of infection and antimicrobial resistance profile. Emphasis is laid on specific patient subpopulations, whose management is discussed.

## 1. Historical Overview of the Emergence and Epidemiology of *Acinetobacter baumannii*: A Significant Pathogen Associated with Nosocomial Infections

*Acinetobacter baumannii* is a Gram-negative, strictly aerobic, coccobacillus mainly associated with hospital-acquired infections. It belongs to the genus *Acinetobacter* which was first recognized in the early 20th century when several microorganisms with the above characteristics were isolated from soil and water [1,2]. In particular, in 1954, Brisou and Prévot proposed for the first time the name *Acinetobacter*—derived from the Greek word “akinetos”, meaning nonmotile—to distinguish nonmotile species from the motile ones of the genus *Achromobacter* [3]. This classification was not broadly recognized until 1968 [4]. *A. baumannii* was first proposed as a new distinct species by Bouvet and Grimont in 1986 using DNA-DNA hybridization [5]. This distinction allowed future clinical and epidemiological studies to focus specifically on *A. baumannii* as an important pathogenic species [2].

*A. baumannii* was initially considered a low-virulence organism and gained clinical interest as a nosocomial pathogen only in the 1970s, as intensive care units (ICUs) and the use of invasive medical equipment became widespread [6]. In contrast to other Gram-negative bacteria, *A. baumannii* can survive on dry surfaces for long periods and form biofilms on abiotic surfaces that allow it to persist in hospital environments, colonize medical equipment and, as a result, spread among critically ill patients and cause recurrent outbreaks [6,7,8,9]. In healthcare environments, transmission is mainly mediated through healthcare workers’ hands, contaminated surfaces, and insufficiently disinfected medical devices [10]. This emerging global health threat was further augmented by the increased use of broad-spectrum antibiotics [2,11].

Most common clinical infections caused by *A. baumannii* include lower respiratory-tract infections, particularly hospital-acquired pneumonia (HAP) and ventilator-associated pneumonia (VAP), with associated mortality ranging from 40% to even 68% in some regions [12,13]. Community-acquired pneumonia (CAP), although infrequent, can progress rapidly with high mortality rates (up to 60% in tropical and subtropical countries such as the Réunion Island, Singapore, and Australia) [14,15,16]. Bloodstream infections represent another clinical challenge, with incidence rates up to 9 per 100,000 population in Israel [17] and 37–40% mortality in the United States and China [18,19]. Moreover, *A. baumannii* frequently colonizes wounds, burns, and surgical sites, especially in trauma, burn and military settings [20]. Lastly, catheter-associated urinary-tract infections are mainly attributed to carbapenem-resistant strains (CRAB), with some studies in India showing 100% resistance to carbapenems [21]. Across all infection types, the persistence of MDR (multidrug) and XDR (extensively drug-resistant) strains, and the emergence of colistin-resistant variants, underscore the urgent need for strengthened infection-control and prevention measures, targeted antimicrobial stewardship, and continuous global surveillance to limit the clinical and epidemiological impact of *A. baumannii* [22].

To highlight the impact of *A. baumannii* infections on individuals’ outcome and public health in general, the World Health Organization (WHO) classified carbapenem-resistant *A. baumannii* as a critical priority pathogen requiring urgent research and new antimicrobial development [11,23].

## 2. Morphological and Biochemical Characteristics of the Pathogen; Mechanisms of Resistance

*Acinetobacter* is a genus comprising many different species, all of which are non-lactose-fermenting, catalase-positive, non-motile, non-fastidious, oxidase-negative, and aerobic Gram-negative coccobacilli [24]. The most recognizable member of the family is *A. baumannii*, due to its higher virulence and more frequent association with hospital-acquired infections, mainly lower respiratory tract infections and catheter-associated bloodstream infections [25]. *A. baumannii* is clearly distinct from the other species of the genus in terms of ecology, pathogenicity and epidemiology. It is associated with significant antimicrobial resistance [26], which is attributed to its ability to form biofilms, mutate porins, lipopolysaccharides (LPS), capsular polysaccharide (CPS), phospholipase (PL), protein secretion systems and two-component efflux pumps [27]. Table 1 summarizes the resistance mechanisms encountered in *A. baumannii* and their clinical significance. A major adaptive defense mechanism of the pathogen is its ability to transform into its mucoid phenotype. This is a variant even more resistant to antibiotics, due to its reduced penetration by them, and presents a barrier against environmental conditions, such as desiccation, disinfection, and immune system recognition [28]. The mucoid phenotype has been recognized in various bacteria as a hypervirulent form (e.g., *Pseudomonas aeruginosa*, *Staphylococcus aureus* and *Klebsiella pneumoniae*), and is characterized by upregulation of the capsular polysaccharide, which is associated both with motility and biofilm formation [29]. Antimicrobial resistance to various classes of antibiotics develops; in XDR strains, highest sensitivity to polymyxins-colistin remains. In a cross-sectional study analyzing carbapenem-resistant *A. baumannii* strains from clinical samples in a large Iranian hospital, >90% were found to be resistant to aminoglycosides. Respectively, almost 100% were resistant to quinolones [30]. Most of the strains encoded for integrase, genes associated with mobile genetic elements, virulence factors, biofilm formation, stress response and host cell death related genes, and antimicrobial resistance genes, mainly β-lactamases [30,31,32]. Its ability to enter a dormant or “persister” state under hostile conditions (e.g., desiccation, nutrient deprivation, antibiotic exposure) further contributes to tolerance to high-dose therapy and may facilitate relapse or chronic colonization.

**Figure 1:**
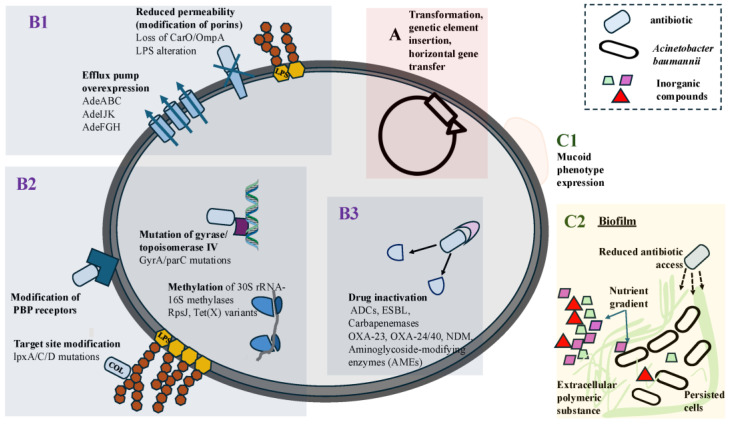
Schematic overview of the main antimicrobial resistance acquisition pathways and resistance mechanisms in *Acinetobacter baumannii*. (**A**) Antimicrobial resistance genes are acquired through transformation, genetic element insertion and horizontal gene transfer. Biochemical and molecular mechanisms of antimicrobial resistance include (**B1**), reduced intracellular drug accumulation due to efflux pump overexpression and decreased outer membrane permeability; (**B2**), target mutation or inactivation (modification of penicillin-binding proteins for β-lactams, lipid A/LPS-lipopolysaccharide modification for colistin, mutations in DNA gyrase/topoisomerase IV for fluoroquinolones, and methylation of 30S rRNA for aminoglycosides); and (**B3**), antimicrobial drug inactivation or degradation (e.g., β-lactamases and aminoglycoside-modifying enzymes). Resistance-associated phenotypes: (**C1**) mucoid phenotype expression and (**C2**) biofilm formation. COL: colistin, PBP: penicillin binding protein, ADCs: Acinetobacter Derived Cephalosporinases, ESBL: Extended Spectrum β-Lactamase, NDM:New Delhi Mutation, OXA, lpx A/C/D, Ade ABC, AdeIJK, AdeFGH, CarO, OmpA, RpsJ, Tet(X): genetic loci.

Resistance in *A. baumannii* rarely arises through a single mechanism. Rather, clones belonging to globally disseminated lineages (e.g., Global Clone 1, Global Clone 2) tend to accumulate multiple resistance mechanisms, such as enzymatic inactivation, target-modification, efflux/porin changes and adaptive persistence, resulting in MDR, XDR and sometimes pandrug-resistant (PDR) phenotypes. Antimicrobial resistance is established through natural transformation (acquisition of mutations, insertion of genetic elements) and/or horizontal gene transfer, in which case, spread is rapidly accelerated [33]. As one review points out, “The typical multidrug resistance phenotype of *A. baumannii* is therefore an orchestrated collimation of all these mechanisms combined with the worldwide spread of “global clones”” [34], summarized in Figure 1.

**Table 1:** Key *Acinetobacter baumannii* resistance mechanisms.

Resistance Mechanism	Representative Examples in *A. baumannii*	Typical Impact	References
β-Lactamase production	OXA-23, OXA-24/40, NDM, ADCs	Carbapenem & cephalosporin resistance	[35,36]
Target mutations/inactivation	gyrA/parC mutations; rpsJ; tet(X) variants; lpxA/C/D mutations	Fluoroquinolone, tigecycline, polymyxin resistance	[37]
Efflux pump over-expression	AdeABC, AdeIJK, AdeFGH	Broad-spectrum reduced susceptibility	[38]
Reduced permeability	Loss of CarO/OmpA, LPS alteration	Lower antibiotic influx, increased tolerance	[39]
Inactivation or reduced binding of aminoglycosides	Production of aminoglycoside-modifying enzymes (AMEs) and 16S rRNA methylases	Aminoglycoside resistance	[36]
Biofilm & persistence phenotypes	Surface adherence, small cell morphotypes	Chronic infection, relapse, colonization	[33,36]

## 3. First-Line Antimicrobials for Carbapenem-Resistant *A. baumannii* Infections

Treatment of MDR *Acinetobacter* remains a significant clinical challenge. In particular, limited therapeutic options exist for the treatment of infections due to carbapenem-resistant *A. baumannii*, at least among conventionally available second-line antimicrobials. Choice of the appropriate antimicrobial agent depends not only on the susceptibility profile of the isolate, but also on the site of the infection and the patient’s profile [40].

According to ECDC, >95% of invasive *A. baumannii* isolates from Greek ICU and non-ICU hospital wards were resistant to carbapenems in 2023 [41]. These statistics clearly place carbapenems outside the scope of reasonable therapeutic use in clinical practice in this setting. A recently published study of carbapenem-resistant *A. baumannii* isolate susceptibility profiling to other classes of antimicrobials in India showed that resistance was similarly high, leaving colistin, minocycline, tigecycline and ampicillin-sulbactam as the last therapeutic resorts, in this order [42]. According to the ECDC report, in Greece, combined resistance of the invasive *A. baumannii* isolates to carbapenems, fluoroquinolones and aminoglycosides exceeded 87% in the most recent recordings. Although antimicrobial synergy against XDR or MDR pathogens has been assessed in several studies, evidence remains limited; consequently, combination regimens are therefore considered empirical/salvage therapies without definitively proven clinical efficacy [43,44].

Colistin remains one of the assets and probably is still the mainstay in the combat against invasive infections from carbapenem-resistant strains. Its expanding use, however, in the treatment of Gram-negative multidrug resistant organisms (MDROs) is already leaving its imprint with high rates of resistance not only in carbapenem-resistant *Klebsiella* strains [45,46] but also among virulent *A. baumannii* isolates [47]. Colistin specifically targets Gram-negative bacteria through electrostatic interactions with phosphate groups on the lipid A moiety of their outer membrane LPS [48]. To a much lesser degree, it acts through the inhibition of respiratory enzymes. Development of resistance in Gram-negative bacteria (GNB), and particularly in *A. baumannii*, has been attributed to modifications of LPS and/or the outer membrane in general, including permeability and efflux pumps [49]. Colistin’s large molecular weight and its cationic properties at normal pH impede its passage through physiological membranes and confine it mainly within the extracellular space [50]. In a retrospective cohort study, treatment failure was not associated with the infected site (pneumonia, bacteremia, urinary tract or other sites of infection), but rather with lower treatment colistin doses [51]. However, higher doses of colistin were associated with higher rates of acute kidney injury, leading to increased mortality. Thus, optimal colistin dose should be adjusted to ideal body weight with close monitoring of both colistin serum concentrations and creatinine levels monitoring [52].

Tetracyclines are bacteriostatic antibiotics with proven efficacy against *Acinetobacter* strains, particularly useful against CRAB. Within this class, two agents show notably greater potency. Minocycline, a semi-synthetic tetracycline derivative, available mainly in oral formulation, have shown in epidemiologic studies in vitro susceptibility against >86% of all *A. baumannii* isolates (when a cut-off MIC of ≤4 mg/L is applied) and in 66% of the CRAB strains [53]. This is due to its ability to overcome resistance mediated through the *Tet* gene encoding efflux pumps [54]. When compared to other members of the class, this translates into 30% improved susceptibility compared to doxycycline and nearly 60% compared to tetracycline. In pharmacokinetic studies, the intravenous formulation has been shown to provide adequate drug exposure for the cure of bacteremia [55], in contrast to the widely prescribed 100 mg BID oral dose; it is therefore preferred over the tablet formulation, especially in combination regimens. Tigecycline, on the other hand, was synthetically designed to overcome this efflux pump-mediated mechanism of resistance and retains activity even if the *TetA* or *TetB* genes are present. Its initially recommended dose (50 mg BID) was soon frowned upon regarding its survival benefit (only 28.6%) for *Acinetobacter*-associated pneumonia [56]. The same review showed significantly increased survival benefit with the double-dose regimen. This high-dose effect was consistent for other infections, such as bacteremias and infections caused by MDROs with concomitant microbiological clearance and similar adverse events. E-testing may overestimate resistance to tigecycline; MIC values still remain <2 mg/L for most isolates, well achievable with the updated dosing recommendation (100 mg BID) of the drug [57]. Eravacycline, the newest addition to the tetracycline class, is a synthetic biocycline, administered both in oral and intravenous formulations, with lower MICs but not fully clarified CLSI (Clinical and Laboratory Standards Institute) breakpoints [58]. Indeed, despite its pharmacokinetic advantages, which enable the drug to achieve higher concentrations in lung tissue with fewer adverse effects, patients infected with *A. baumannii* had lower pathogen clearance and higher mortality rates [59]. As such, its clinical use is still under investigation.

Sulbactam is both an Ambler Class A β-lactamase inhibitor as well as a β-lactam with intrinsic antibacterial activity against *Acinetobacter* species, mainly due to inhibition of penicillin-binding proteins 1 and 3 (PBP1 and PBP3). Although true susceptibility to sulbactam ranged from 26.2% to 56.0% in an epidemiological study recently published from the *Acinetobacter* strains maintained in the National Biorepository, a series of meta-analyses have shown a very important survival benefit of patients treated with high-dose ampicillin sulbactam, either as stand-alone or in the context of a combination regimen, even if the actual in vitro resistance rate was significant [56,60]. Based on these studies, IDSA recommended high-dose sulbactam treatment as backbone therapy for serious CRAB-induced infections [61]. Sulbactam-durlobactam, a novel combination of sulbactam and next-generation diazabicyclooctane (DBO) β-lactamase inhibitor durlobactam, was recently approved by the FDA in 2023 for pneumonias caused by *Acinetobacter* strains. Durlobactam represents a DBO β-lactamase inhibitor, which can restore sensitivity to sulbactam, by lowering its MIC [62]. The ATTACK trial, a phase-3 multicentric study aimed to compare the efficacy and safety of sulbactam-durlobactam versus colistin in combination with a carbapenem for the treatment of patients with serious infections caused by *A. baumannii*, showing non-inferiority and reduction in mortality of sulbactam-durlobactam to its comparator in the case of HAP/VAP [63]. Its efficacy is questionable in bloodstream infections and real-world experience with the drug remains sparse. As such, current guidelines advise against its use in the case of available active, less potent antimicrobials.

Recently, cefiderocol, a broad-spectrum β-lactam, active against carbapenem resistant Enterobacteriaceae (CRE), XDR *Pseudomonas* and *XDR A. baumannii*, among others, with a novel iron-carrier mechanism of action, was developed and approved for use in infections caused by MDRO Gram-negative pathogens. In the conducted clinical trials, higher mortality was recorded in CRAB infections treated with cefiderocol; however, this was later attributed to higher corresponding patient severity. Both ESCMID and IDSA recommend its use only as second-line, salvage treatment, preferably combined with another drug [64,65]. Meta-analyses remain controversial and based mainly on observational studies. They show better survival with increased doses of cefiderocol [66]. Increasingly reported heteroresistance/resistance is also a concerning matter. Main treatment modalities for XDR *Acinetobacter* and their mechanisms of action are summarized in Table 2.

**Table 2:** Clinically assessed treatment options for systemic CRAB infections.

Drug	Class/Mechanism of Action	Indication/Type of Infection	Special Considerations	References
Colistin	Polymyxins/interaction with lipid A moiety of outer membrane	HAP/VAP/ bacteremia/UTI	Nephrotoxicity	[48,49,50,51,52]
Minocycline	Tetracyclines/interaction with 30S ribosome, inhibition of protein synthesis	Pneumonia/CNS/prostate/abdominal infections	Gastrointestinal adverse effects/photosensitivity	[53,54,55]
Tigecycline	Tetracyclines/interaction with 30S ribosome, inhibition of protein synthesis	Bile duct/abdominal infections	Gastrointestinal adverse effects/ hepatic dysfunction	[56,57]
Eravacycline	Tetracyclines/interaction with 30S ribosome, inhibition of protein synthesis	Complicated intra-abdominal infections	Gastrointestinal adverse effects/photosensitivity	[58,59]
Sulbactam	Beta-lactamase inhibitor	Skin and skin structure infections, intra-abdominal/gynecological infections, HAP/VAP	Gastrointestinal adverse effects	[56,60,61,62,63]
Cefiderocol	Inhibition of PBP3-cell wall synthesis/binding to outer membrane iron transporters	Urinary tract, HAP/VAP and systemic infections	Gastrointestinal adverse effects, hepatotoxicity	[64,65,66]

## 4. Combination Treatment vs. Monotherapy: Evidence from Existing Literature

Due to the paucity of available antimicrobial agents with potent activity against XDR and PDR pathogens, suggested treatment modalities may employ combination regimens rather than monotherapy. In the case of *A. baumannii*, therapeutic drug levels with the few treatment alternatives left in the case of carbapenem resistance are difficult to achieve, especially in the presence of comorbidities. Close drug level monitoring is recommended to ensure avoidance of the development of adverse effects, mainly acute kidney injury, especially in the case of colistin, one of the backbone treatments. Additionally, available mainstay treatments in analogous cases are frequently associated with potential adverse effects (neurological complications of colistin, hepatotoxicity and autoimmune hypersensitivity reactions of tetracyclines, etc.). Whenever possible, and if not otherwise contraindicated, critically ill patients are usually treated with at least two active antimicrobials, or even though data may not be robust, with combination regimens of antibiotics to which the isolates may exhibit in vitro resistance. A previously published meta-analysis of 10 studies (three RCTs and seven retrospective studies) showed superiority of the combination treatment of meropenem with colistin over colistin monotherapy for carbapenem-resistant *A. baumannii* strains [67]. Although the exact mechanism of synergism remains unclear, it has been suggested that the effect could be attributed to the permeabilizing effect of colistin on the bacterial outer membrane, permitting the entry of large hydrophobic molecules. In this analysis, adverse effects did not differ significantly between the two groups. However, the OVERCOME trial, a double blind, randomized, polycentric study, aimed to directly compare these two treatment options, and contradicted these findings showing that, when colistin dosing was strictly adjusted to the ideal body weight, outcome was similar in-resistant *Acinetobacter* infections, even in the case of pneumonia [68]. The combination most used in clinical practice, typically for carbapenem-resistant *Acinetobacter* strains, and when no other treatment options are available, is colistin with tigecycline, at least for cases demonstrating in vitro susceptibility to these agents [69]. However, robust clinical data supporting this regimen remain scarce and insufficiently analyzed.

The beneficial effect of adding ampicillin–sulbactam to the treatment of patients with *Acinetobacter* infections does not seem to depend strictly on confirmed in vitro susceptibility of the strains. In several studies, administration of high dose sulbactam (6–9 gr per day) regardless of the formulation (ampicillin/sulbactam, sulbactam, or cefoperazone/sulbactam) resulted in significant improvement of the patients’ clinical outcome, even when most of the isolates were proven to lack susceptibility to the drug [70]. This effect appears to be even better established in nosocomial lower respiratory tract infections [71]. Clinical reports exist on combinations of sulbactam mainly with colistin, a tetracycline (tigecycline or minocycline) or levofloxacin [60,72].

Fosfomycin is the single compound of its antibiotic class; it is a low-molecular weight compound which interferes with cell wall synthesis through inhibition of peptidoglycan biosynthesis. Even though it was previously considered a simple oral antibiotic for the treatment of mild, uncomplicated urinary tract infections in women, in the era of antimicrobial resistance, its utility was rediscovered initially in the treatment of difficult-to-treat infections caused by Gram-negative bacteria with its intravenously administered formulation [73]. In vitro studies suggested synergy of fosfomycin with other antimicrobials for bacterial clearance both in the case of susceptible [74] or non-susceptible strains [75]. In the case of carbapenem-resistant *A. baumannii*, despite the bacterial inherent resistance to fosfomycin, its addition to a carbapenem improved inhibition of bacterial growth in vitro [76]. In the small-scale clinical studies reported in the literature, combination of colistin with fosfomycin resulted in microbiological and clinical improvement [77,78]. Similarly, intravenous fosfomycin has also been combined with cefiderocol and ampicillin–sulbactam with a more favorable outcome in the cefiderocol-containing regimens [79,80].

Other antimicrobials with reported synergistic activity against resistant *A. baumannii* strains include rifampicin usually in combination with colistin (particularly when used to treat pneumonia and/or central nervous system infections) [81,82,83]. Promising in vitro results were recorded when colistin was combined with antibiotics that exclusively target the cell wall/membrane of Gram-positive bacteria (glycopeptides-vancomycin, lipopeptides-daptomycin). This effect has theoretically been attributed to the lack/mutated forms of LPS in drug-resistant *Acinetobacter* strains. Moreover, survival of *Galleria mellonella* larvae infected with *Acinetobacter* was significantly enhanced in the colistin–vancomycin combination compared to colistin monotherapy [84]. There is limited data regarding the implementation of these treatment strategies in clinical practice with questionable clinical benefit and considerable safety issues [85]. A summary of most clinically relevant combinations treatment strategies for XDR *A. baumannii* can be found in Table 3.

**Table 3:** Proposed synergistic combination treatments for XDR *A. baumannii*.

Drugs	Proposed Mechanism of Action	Type of Infection Used	Strength of Available Data	References
Colistin–meropenem	Permeabilization of the bacterial outer membrane, permitting the entry of large hydrophobic molecules (carbapenem)	Bacteremia, HAP/VAP	Low	[67,68]
Colistin–tigecycline	Disruption of the membrane facilitating penetration of tigecycline in the cell	Bacteremia, HAP/VAP	Low	[69]
Colistin–glycopeptides	Disruption of the outer membrane, enabling glycopeptides to access cell wall targets from which they are usually excluded	In vitro data	Low	[84,85]
Colistin–daptomycin	Colistin disrupts the bacterial outer membrane, allowing daptomycin to reach its target inside the cell (cytoplasmic membrane)	In vitro data	Low	[84,85]
Sulbactam–colistin	Both drugs’ act against bacterial cell components, particularly the cell envelope	Mainly HAP/VAP	Moderate	[60,70,71,72]
Sulbactam–minocycline	Minocycline can overcome certain resistance mechanisms, such as efflux pumps (e.g., TetA and RND pumps) and thus enhance the function of the bata-lactamase inhibitor	In vitro data HAP/VAP	Moderate	[60,70,71,72]
Sulbactam–tigecycline	Sulbactam may reduce the MIC of tigecycline	HAP/VAP	Low	[60,70,71,72]
Fosfomycin–colistin	Synergistic inhibition of synthesis and disruption of the membrane	HAP/VAP, Bacteremia, SSTI, Intra-abdominal	Moderate	[77,78]
Fosfomycin–cefiderocol	Fosfomycin weakens the bacterial cell wall, and cefiderocol can more effectively deliver enter and bind to its target	HAP/VAP, Bacteremia, CVC infection	Moderate	[74,75]

## 5. Treatment Considerations Based on Patients’ Characteristics/Types of Infections

Management of resistant *Acinetobacter* infections, while inherently puzzling, is further complicated in certain patient groups, where antibiotic selection and dosing require extra caution. These special patient populations often require tailored antimicrobial strategies due to altered pharmacokinetics, increased risk of drug toxicity and limited safety data for many last-line agents (Table 4). Thus, optimizing therapy in these situations requires a careful balance between efficacy and safety, guided by susceptibility testing, individualized dosing and multidisciplinary clinical judgment.

### 5.1. Biofilms

Biofilms play a central role in the persistence of *A. baumannii* in hospital environments and patients. These microbial communities protect bacteria from antibiotics and immune defenses, rendering infections notoriously difficult to eradicate. Biofilm formation occurs on endotracheal tubes, catheters, prosthetic devices, and wounds. Within biofilms, bacteria exhibit slower metabolic activity, limiting the efficacy of conventional antibiotics. Additionally, the biofilm matrix restricts antibiotic penetration and facilitates horizontal gene transfer, promoting multidrug resistance [86].

Epidemiological data from critically ill patients identify *A. baumannii* as one of the most frequent central-line associated bloodstream infection (CLABSI) pathogens [87]. Current clinical guidelines, including those by the Infectious Diseases Society of America (IDSA), strongly recommend the immediate removal of the indwelling catheter as the primary therapeutic intervention. Retention of the catheter in the setting of *Acinetobacter* bacteremia is independently associated with treatment failure, persistent bacteremia, and increased mortality rates [88]. In selected cases where catheter removal is deemed not feasible (e.g., limited vascular access), catheter salvage may be attempted using Antibiotic Lock Therapy (ALT) as an adjunct to systemic treatment. Another novel therapeutic avenue is bacteriophage therapy, and current evidence supports that combination of phages and antibiotics could increase biofilm eradication and provide new insight into the treatment of biofilm-associated infections caused by antibiotic-resistant bacteria [89]. A handful other options including quorum-sensing inhibitors and biofilm-disrupting agents, such as N-acetylcysteine [90] or EDTA [91], show promise in laboratory and early clinical studies, but further trials are needed to establish standardized dosing and safety.

Colistin, tigecycline and levofloxacin have shown significant antibiotic in vitro activity against *Acinetobacter baumannii* biofilms, while antibiotic combinations with rifampicin or clarithromycin have demonstrated synergistic effects [92]. In addition, several proposed biofilm inhibitors (zinc lactate, stannous fluoride, furanone, azithromycin, rifampicin) in combination with conventional antibiotic regimens, such as imipenem, meropenem, tigecycline and polymyxin B, have demonstrated in vitro synergy against biofilm-forming carbapenem-resistant strains [93] in early clinical studies, but further trials are needed to establish standardized dosing and safety.

### 5.2. Pneumonia

Carbapenem-resistant *A. baumannii* pneumonia represents a major clinical challenge due to limited therapeutic options and high mortality. Recent advances have focused on sulbactam-based combinations, particularly sulbactam-durlobactam. Both these drugs have a sufficient penetration into epithelial lining fluid (ELF) (86% for sulbactam and 41.3% for durlobactam) [94]. In the pivotal ATTACK phase-3 trial, sulbactam-durlobactam demonstrated non-inferior efficacy and markedly lower nephrotoxicity compared with colistin for serious *A. baumannii-calcoaceticus* complex infections, with a 28-day mortality of 19% versus 32% in the colistin arm. All the patients were concomitantly under imipenem–cilastatin [68]. Accordingly, this regimen could be a good option for confirmed or suspected CRAB pneumonia where available.

Cefiderocol exhibits potent in vitro activity against multidrug- and colistin-resistant *A. baumannii*. Nonetheless, its penetration into epithelial lining fluid (ELF) is suboptimal [95,96]. Real-world data from clinical trials remain variable. In the “Efficacy and safety of cefiderocol or best available therapy for the treatment of serious infections caused by carbapenem-resistant Gram-negative bacteria” (CREDIBLE-CR) trial, higher all-cause mortality was observed among CRAB infections treated with cefiderocol compared with best available therapy, particularly in patients with nosocomial pneumonia or bloodstream infection or sepsis [97]. Differences in all-cause mortality were not observed in the APEKS-NP study, where patients with Gram-negative nosocomial pneumonia—including *A. baumannii* pneumonia—were randomized to either cefiderocol or extended-infusion meropenem. It should be noted, however, that this study also included meropenem non-susceptible isolates, indicating that cefiderocol was non-inferior to a non-active agent [98]. In another meta-analysis comparing cefiderocol and colistin-based regimens for the treatment of severe CRAB infections, the cefiderocol-based group was associated with lower all-cause mortality, but not in ventilator-associated pneumonia (VAP) patients [99].

High-dose tigecycline provides an adjunct option for CRAB pneumonia, particularly when there are limited therapeutic options. Indeed, increased tigecycline doses lead to increased ELF penetration, supporting its use in VAP [100]. Tigecycline monotherapy is associated with higher mortality rates, whereas several observational studies suggest that high-dose tigecycline regimens, combined with other agents, especially colistin or high-dose ampicillin–sulbactam, may improve treatment outcomes in CRAB pneumonia [101,102]. Regarding other tetracycline derivatives, minocycline concentrations in ELF were found to exceed concentrations in serum by at least 2-fold [103]. Data from a retrospective study using minocycline in combination with other agents against MDR *A. baumannii* infections (including 58% pneumonias) demonstrated clinical success in 73% of cases, suggesting minocycline as a potentially effective option for CRAB pneumonias, when used as combination regimen [104]. Eravacycline, has been shown to achieve ELF concentrations 6-fold higher than serum levels in healthy adults [105]. However, a retrospective study including 93 patients with MDR A. baumannii pneumonia reported increased mortality among those treated with eravacycline, although these patients more frequently presented with *A. baumannii* bacteremia and co-infection with severe acute respiratory syndrome coronavirus-2 (SARS-CoV-2) [59]. Current IDSA guidance suggests limiting its use in situations where other agents are unavailable or inactive [65]. The activity of intravenous colistin in ELF is suboptimal, raising concerns about its use on pneumonia by resistant organisms [106]. Current IDSA recommendations support the use of colistin in combination with at least one other agent for CRAB infections. However, clinical trials have not demonstrated statistical significance benefits for combining colistin with rifampicin, fosfomycin, or meropenem compared to monotherapy [65,68,79,82,107]. To date, only one clinical trial including 39 patients with CRAB pneumonia has shown a clear advantage for combination therapy of colistin with high dose of ampicillin–sulbactam compared to colistin monotherapy [62]. Inhaled colistin as adjunctive therapy for CRAB pneumonia is currently not recommended due to lack of data demonstrating a clear beneficial effect [65,108,109].

### 5.3. CNS Infections

*A. baumannii* can cause hospital-acquired meningitis and ventriculitis following trauma, external ventricular drainage, or shunt placement in neurosurgical patients. These infections pose major therapeutic challenges because XDR *A. baumannii* has limited antibiotic options and most agents have poor cerebrospinal-fluid (CSF) penetration. Colistin, once the last-line therapy, is now undermined by emerging resistance. Management relies on aggressive source control (removal or replacement of external ventricular drains or shunts) combined with systemic and, when possible, intraventricular/intrathecal (IVT/ITH) antimicrobial therapy.

Therapeutic options are limited but evolve. Colistin, which represents the cornerstone of CRAB infections management, has poor penetration into CSF. For CNS infections, IVT/ITH colistin as an adjunct to intravenous (IV) colistin is associated with lower mortality rates [110]. Even when colistin resistance is present, IVT colistin in combination with IVT tigecycline, IV colistin and IV tigecycline—which has also limited penetration into the CNS—has successful treatment outcomes [111,112,113]. Additionally, use of IVT aminoglycosides with IV colistin or carbapenems has been shown to achieve clinical cure in 79% of the cases of *Acinetobacter* meningitis involved [114]. However, IVT/ITH administration of antibiotics should be performed with caution, as it could cause meningeal irritation [110].

Newer agents have shown promising results, but data are sparse and limited to case reports. Cefiderocol, used as part of combination therapy, has been associated with successful outcomes in CRAB meningitis/ventriculitis [115,116,117]. More specifically, cefiderocol combined with high-dose ampicillin/sulbactam in a patient with CRAB ventriculitis, or with intraventricular gentamicin in another patient with CRAB meningitis, resulted in microbiological and clinical cure [115,116]. Adequate CSF penetration of 60 and 68% of plasma AUC has been demonstrated, when given at a dosage of 2 g every 6 or 8 h, respectively. However, published data indicate that cefiderocol exhibits considerable heterogeneity in CSF pharmacokinetics (ranging from 4% to 68%), likely influenced by meningeal inflammation and dosing strategy [116,118,119,120]. Sulbactam-durlobactam has also achieved successful outcomes in recent case reports of CRAB meningitis or ventriculitis, in combination with meropenem, cefiderocol, or cefiderocol and minocycline [61,121,122]. Minocycline, as a lipophilic agent, exhibits good penetration into the CSF, regardless of meningeal inflammation [123].

### 5.4. Endocarditis

Although infective endocarditis is most commonly caused by Gram-positive organisms, Gram-negative pathogens—including *A. baumannii*—can rarely cause endocarditis, with high mortality rates [124,125]. In a series of 35 studies with *Acinetobacter*-associated endocarditis, a prosthetic valve was present in 40.5%, and the aortic valve was the commonest infected site, followed by mitral valve [124]. Diagnosis was set with transthoracic echocardiography in 48.6%, and at autopsy in 20%. The rise of XDR *A. baumannii* introduces considerable therapeutic challenges. Effective therapeutic options are further reduced since antimicrobial penetration into vegetations is limited due to biofilm formation. In an experimental animal model of *A. baumannii* endocarditis, colistin was effective on bacterial clearance from blood but not from vegetations [126].

Data for CRAB endocarditis treatment are limited to case reports and systematic reviews. In one of these reviews including 37 patients with infective *A. baumannii* endocarditis (66.7% carbapenem-resistant), aminoglycosides, cephalosporines and carbapenems were the commonest antimicrobials used [124]. In another review, 26 patients with infective endocarditis due to carbapenem-resistant Gram-negative bacteria, including *A. baumannii* (19.2% of the pathogens), were mainly treated with aminoglycosides, cephalosporins, carbapenems and colistin. Surgical management along with antimicrobial therapy was performed in 53.8% of the cases [125]. Although promising data exist regarding treatment of other XDR *A. baumannii* infections with novel agents, such as sulbactam-durlobactam and cefiderocol, no data exist on infective endocarditis. Reported management is guided by the microbiological susceptibility profile of the strain, and usually includes combination therapy, as monotherapy has poor outcomes, while early surgical intervention (valve replacement or repair) may be associated with improved survival in reported cases [127].

### 5.5. Chronic Kidney Disease (CKD)

Carbapenem-resistant *Acinetobacter* infections in CKD patients pose unique pharmacological and safety challenges. Renal dysfunction alters antibiotic pharmacokinetics, possibly resulting in subtherapeutic levels or drug accumulation, while proposed loading doses remain unaffected.

Among the agents used, colistin presents the highest risk for nephrotoxicity and therefore needs dose adjustment in renal impairment, with possible detrimental effects especially in CKD patients [128]. It frequently causes reversible acute tubular necrosis (average incidence 25%) due to localization of drug in proximal tubular cells and acts as an aggravating modality in patients with further risk factors for nephrotoxicity, such as extreme age, obesity, diabetes, hypertension and concomitant exposure to nephrotoxins. Daptomycin, while not inherently nephrotoxic, requires monitoring of CPK levels due to risk of rhabdomyolysis-induced renal injury with dose adjustment in CrCl < 30 mL/min. Ampicillin/sulbactam and meropenem can cause acute interstitial nephritis, while fosfomycin exhibits a low nephrotoxic potential, all three of them also in need of dose-adjustment. Tetracyclines, namely minocycline and tigecycline, are mainly hepatically eliminated (~10–15% renal elimination), making them an attractive option irrespective of renal function levels. Novel agents, cefiderocol and sulbactam/durlobactam, do require dose adjustment in renal impairment [129,130], but generally exhibit minimal renal toxicity.

In critically ill CKD or dialysis patients, therapeutic drug monitoring (TDM) is crucial. Maintaining optimal drug exposure is essential, as subtherapeutic concentrations can promote resistance and treatment failure. After hemodialysis, supplemental antibiotic doses may be necessary to compensate for drug removal. A review on pharmacokinetic studies including critically ill patients on hemodialysis and continuous renal replacement therapy showed highly variable drug exposure and target attainment rates. Based on these conclusions, the authors suggest therapeutic drug monitoring for aminoglycosides, beta-lactams, glycopeptides, linezolid, and colistin and recommend it for daptomycin, fluoroquinolones, and tigecycline to optimize outcome and deter adverse effects [131]. Adjunct strategies, such as combination therapy with non-nephrotoxic agents (e.g., cefiderocol plus minocycline) [132], and supportive care measures like avoiding other nephrotoxic drugs (aminoglycosides, vancomycin) are key to optimizing outcomes.

### 5.6. Pregnancy

Another exceptionally challenging group of patients with carbapenem-resistant *A. baumannii* infections are pregnant women, since they usually do not meet the inclusion criteria of most clinical trials and, as a result, there are limited safety data for most active antibiotics in this population [133].

Polymyxins (colistin, polymyxin B) are classified as pregnancy category C by the FDA because of teratogenic and embryotoxic effects observed in animal studies. As a result, given also the approval of novel agents against *Acinetobacter*, the use of polymyxin therapy is downshifted as a last line option in case of obvious benefit outweighing the risk. When no safer alternatives exist, colistin can occasionally be cautiously used in late pregnancy with monitoring for maternal and fetal toxicity. Tigecycline is classified as a pregnancy category D antibiotic, as it can cross the placenta and affect fetal bone and tooth development, making it generally contraindicated. Cefiderocol and sulbactam/durlobactam have minimal clinical data in pregnancy but have not shown significant teratogenicity in animal studies (pregnancy category B), suggesting potential as last-resort agents with possibly safe use. When facing MDR isolates of *A. baumannii* and seeking antibiotic synergy through various antibiotic combinations, the use of beta-lactams (ampicillin/sulbactam, meropenem), fosfomycin (data only for first trimester) and daptomycin, all categorized as pregnancy category B, provides another alternative [134].

### 5.7. Pediatric Patients

When dealing with carbapenem-resistant *A. baumannii* in infants and children, therapeutic options become even more complex than in adults, given the limited pharmacokinetic, safety and efficacy data in this age group. Although colistin has been used in pediatric settings for infections due to multidrug-resistant Gram-negative bacteria including *A. baumannii*, standard dosing in children remains largely extrapolated from adult data and small case series. Currently recommended doses by FDA and EMA of 75,000–150,000 IU/kg/day may lead to suboptimal exposure, resulting in plasma concentrations < 2 mg/L, which is the breakpoint for susceptibility of nosocomial Gram-negative pathogens to colistin. A population pharmacokinetic study suggested that administration of colistin in higher doses of 200,000–350,000 IU/kg/day for infections caused by carbapenem-resistant Gram-negatives resulted in improved exposure and is well-tolerated [135].

In a comparable manner to adults, current guidelines for severe infections caused by resistant *A. baumannii* in children generally advise against the use of colistin monotherapy [93]. In cases where a second in vitro active agent is available and appropriate for the site of infection, combination therapy is preferred [136]. Most published pediatric case series involve colistin-susceptible *A. baumannii*, and successful treatment regimens combining colistin with agents such as tigecycline or ampicillin/sulbactam have been reported. Notably, a case series of neonates with extremely drug-resistant (XDR) *A. baumannii* sepsis reported successful clinical cure using a combination of colistin, tigecycline and cefoperazone/sulbactam, achieved through careful dosing and close monitoring [137]. Regarding tigecycline dose, according to a case series of 13 critically ill children with infections caused by XDR Gram-negative pathogens (including *A. baumannii*), a loading dose of 1.8–6.5 mg/kg followed by 1.0–3.2 mg/kg q12h was well-tolerated and resulted in good clinical response [138]. Sulbactam-containing regimens have also demonstrated superior efficacy compared to alternative regimens with decreased mortality in children suffering from A. baumannii bacteremia [139]. Suggested dose of ampicillin/sulbactam is 400 mg/kg/day (per ampicillin component) divided every 4 to 6 h, with extended infusion over 4 h [140]. Last but not least, while cefiderocol is adequately studied for CRAB infections mostly in adult populations, promising—though limited—data support clinical effectiveness also in children at doses of 60 mg/kg/dose every q8h [140].

## 6. Novel Antimicrobial Agents Currently in the Development Pipeline

### 6.1. Antimicrobial Drugs

Cefepime/zidebactam is a fourth-generation cephalosporin combined with a non-beta lactam/beta-lactamase inhibitor targeting the synthesis of the peptidoglycan layer. Zidebactam belongs, together with avibactam and relebactam, to a new β-lactamase inhibitor category, the diazabicyclooctanes. It binds with high affinity to penicillin-binding protein 2 (PBP2) and inhibits β-lactamases, thereby preventing hydrolysis of cefepime and enhancing its antimicrobial activity [141]. Although its in vitro antimicrobial activity against *A. baumannii* is moderate, the combination in vivo augments cefepime’s pharmacodynamics and has been shown to effectively reduce the bacterial load in the lung and thigh of infected neutropenic mice [142,143]. It is currently investigated in phase-3 clinical trials, with carbapenems as comparators.

Imipenem/cilastatin/funobactam is a novel combination of imipenem with funobactam, a serine-β-lactamase inhibitor against β-lactamases of Class A, C, and D. It has shown efficacy in infections caused by carbapenem-resistant *Acinetobacter* and *Klebsiella* spp. in murine in vitro and in vivo studies and is currently being evaluated in phase-3 urinary tract infection and hospital-acquired pneumonia clinical trials [144].

### 6.2. Bacteriophages

Phage therapy dates to 1919. The discovery of penicillin and the initiation of the era of antibiοtic treatment, however, set its use aside, up until recently, with the emergence of the multi drug-resistant ESKAPE (*Enterococcus faecium*, *Staphylococcus aureus*, *Klebsiella pneumoniae*, *A. baumannii*, *Pseudomonas aeruginosa*, and *Enterobacter* spp.) pathogens. Lytic phages, single or in cocktail formulations, can kill host bacterial cells without affecting the non-pathogenic microbiome and can act synergistically with antimicrobials (phage antibiotic synergy) [145]. Bacteriophages exhibit two types of life cycle; the lytic and the lysogenic one. During the lytic cycle, phages enter the bacterial cell, through binding to specific receptors and their viral genome enters the bacterial cell. The phage then takes over the infected cell, leading to production of new phage particles, which are released via cell burst. In the lysogenic cycle, phage DNA integrates with the host bacterial genome and remains in a dormant state (prophage). Switch can occur to the lytic cycle under specific conditions [146]. Phage enzymes may additionally separately contribute to bacterial host killing. Bacteriophages have high host specificity. Μany phages, specifically targeting *Acinetobacter* spp., have already been sequenced and characterized [147]. Until now, no phage-based therapies have been approved by regulatory authorities. Anecdotal cases of successful implementation of *Acinetobacter* specific phages have been reported [148]. As phages co-evolve with bacteria in favor of bacterial survival, the issue of bacterial survival, phage resistance development through, e.g., modification of bacterial surface receptors, and/or changes in fitness and virulence of bacteria induced by phage invasion may affect microbiological and clinical response; in this sense phage combination is recommended [149].

### 6.3. Antimicrobial Peptides

Antimicrobial peptides (AMPs) are short, often positively charged peptides that may directly mediate lysis of the bacterial membrane. They occur in nature as part of the intrinsic defense mechanisms of various organisms, modulating the host’s immune system [150]. Important advantages are the retaining of efficacy of the antimicrobial peptides regardless of the pre-existence or development of pathogen resistance, and potential synergy either with other antimicrobial peptides and/or antimicrobials [151]. Octopromycin, a positively charged, largely hydrophobic, proline-rich product of gene 5 of *Octopus minor*, has shown significant in vitro elimination of persister *Acinetobacter* cells, leading to the destruction of biofilms [152]. Pap12-6-10, a 12-mer peptide derived from the N-terminus of papiliocin modulates LPS-induced inflammatory responses. In a carbapenem-resistant *A. baumannii*-induced sepsis mouse model, Pap12-6-10 protected organ damage from septic shock and displayed significant therapeutic effects without significant cytotoxicity [153]. Similarly, biotechnologically produced peptides have also shown potent in vitro cidal activity against *Acinetobacter* strains [154]. Treatment strategies implicating AMPs appear promising. However, challenges that still remain in their path to clinical application are potential cytotoxic effects, production costs, and problems related mainly to peptide bioavailability [155].

## 7. Summary of Current State: Where Do We Stand

In our review of the published data on treating XDR *A. baumannii*, we emphasized that antimicrobial resistance represents a major health threat globally; it currently accounts for roughly 5 million deaths annually worldwide, estimated to increase to 10 million deaths in 2050. Carbapenem-resistant *A. baumannii* is one of the leading pathogens in this list of antimicrobial-resistant pathogen burden (ranking roughly fourth), whose greatest impact is inflicted upon low-resource countries. Associated mortality, especially with resistant strains, is reported to exceed 40% [156].

Treatment of *Acinetobacter*-attributed infections is challenging. In general, in the case of multi-drug-resistant Gram-negative bacteria (usually defined as carbapenem-resistant Gram-negative pathogens), it has been known that monotherapy is associated with higher mortality, lower clinical success and lower microbiological eradication. Although in vitro synergy has been shown in several studies, the in vitro findings do not always align with real-world data (e.g., AIDA trial, [157]). The combination treatment-related clinical benefit is usually more pronounced in the context of at least one active antimicrobial in the regimen, usually a beta-lactam. In the case of resistant *Acinetobacter*, however, a systematic review and metanalysis of published clinical studies including at least 10 patients each, and evaluating both clinical and microbiological response, concluded that consensus has still not been reached [158]. In our current review we found that although monotherapy was inferior in general in infections with resistant carbapenem resistant GNB compared to combination treatments, for CRAB infections, no significant differences were observed in mortality, clinical success and microbiological eradication. Of note, microbiological clearance was also comparable when ceftazidime–avibactam was used, an antibiotic of which resistance rates exceeding 50% of *Acinetobacter* strains are recorded [159]. The same differentiating observation was recorded for clinical response in the case of *Acinetobacter* in contrast to CRE pathogens (OR 1.15 vs. 1.5). Recent meta-analyses of studies for the treatment of severe infections caused by carbapenem-resistant *Acinetobacter baumannii* have further confirmed these findings; monotherapy was associated with similar rates of treatment success with no statistical difference in terms of safety [99]. This observation may not, however, truly reflect applicability to all patients involved. Most available studies are observational, not actually completely ruling out the treating physicians’ bias, and could have directed “sicker” patients to the combination treatment cohort. In any case, it is probably more accurate to say that “the jury is still out”. The design of blinded, randomized studies on a large scale will be able to objectively address this clinical issue. Obviously, any review of published data suffers from the problems of the original data and the limitations of original studies.

The historical evolution of *A. baumannii* illustrates the dynamic interplay between microbial adaptation, hospital environments, and clinical management. This species managed to transform from an environmental organism into a perilous pathogen responsible for significant morbidity and mortality worldwide. Its epidemiological success underscores the importance of continuous surveillance, infection control, and stewardship to prevent further catastrophic consequences, as pharmaceutical treatment modalities are limited. To date, *Acinetobacter* remains a global public health concern causing invasive infections that remain challenging to the treating physician, especially in sub-cohorts of patients with co-morbidities or other unique characteristics.

## Figures and Tables

**Table 4:** Considerations of treatment for special types of infection/populations.

	Proposed Regimens	Special Considerations
Biofilms	a. Core antibiotics: Imipenem, meropenem, tigecycline and polymyxin B. b. Future therapeutic options: Quorum-sensing inhibitors and biofilm-disrupting agents (e.g., N-acetylcysteine, EDTA, etc.), bacteriophages.	a. Mechanical interventions. b. Consider combination with biofilm inhibitors (zinc lactate, stannous fluoride, furanone, azithromycin, rifampicin).
Pneumonia	a. Sulbactam-based combinations. b. Cefiderocol-based regimens. c. High-dose tigecycline combination regimens. d. Colistin synergistically with other antimicrobials.	Sub-optimal levels of most active antimicrobials in ELF.
CNS infections	a. First-line treatment: Colistin IV and IVT/ITH. b. Cefiderocol combination regimens. c. Minocycline.	a. Aggressive source control together with combination of systemic and intraventricular/intrathecal therapy. b. Limited penetration of standard of care antimicrobials in the CNS. c. IVT/ITH administration of antibiotics associated with meningeal irritation.
Endocarditis	Strictly combination regimens; limited clinical data.	Early surgical intervention is highly recommended.
CKD		a. Therapeutic drug monitoring is crucial to maintain optimal drug exposure (colistin, aminoglycosides, vancomycin). b. Supplemental antibiotic doses after hemodialysis may be required. c. Combination of active antimicrobials with other nephrotoxic drugs is discouraged.
Pregnancy	a. The majority of active antibiotics exhibit limited safety data in this population (patients are excluded from clinical studies). b. Sulbactam- and cefiderocol-based regimens (no evidence of teratogenicity in animal studies-pregnancy category B).	a. Antibiotic therapy should use the lowest effective dose and shortest duration possible, guided by microbiological data and obstetric consultation. b. Strict infection control measures are essential to prevent outbreaks.
Pediatric patients	a. Limited pharmacokinetic, safety and efficacy data in this age group. b. Colistin remains the standard-of-care with limited pharmacokinetic data. c. In case of colistin resistance: Sulbactam-based regimens, tigecycline (although off-label in younger children and limited by pharmacokinetics) and novel agents or compassionate use antibiotics if available.	a. Optimize supportive care (source control, removal of infected devices, ventilator/line management). b. Close infectious disease and pharmacy consultation are essential due to the increased risk of further resistance development.

## Data Availability

The data presented in the present review is review of published data. No new data were created or analyzed in this study.

## Abbreviations

The following abbreviations are used in this manuscript:

ALT	Antibiotic Lock Therapy
AMP	Antimicrobial Peptide
BID	Twice A Day
CAP	Community-Acquired Pneumonia
CDC	Centers for Disease Control and Prevention
CKD	Chronic Kidney Disease
CLABSI	Central-Line Associated Bloodstream Infection
CLSI	Clinical and Laboratory Standards Institute
CNS	Central Nervous System
CPK	Creatine Kinase
CPS	Capsular Polysaccharide
CRAB	Carbapenem-Resistant *Acinetobacter baumannii*
CrCl	Creatinine Clearance
CRE	Carbapenem-Resistant Enterobacteriaceae
CSF	Cerebrospinal Fluid
DBO	Diazabicyclooctane
ECDC	European Centre for Disease Prevention and Control
ELF	Epithelial Lining Fluid
EMA	European Medicines Agency
ESCMID	European Society of Clinical Microbiology and Infectious Diseases
FDA	Food and Drug Administration
GNB	Gram-negative Bacteria
HAP	Hospital-Acquired Pneumonia
ICU	Intensive Care Unit
IDSA	Infectious Diseases Society of America
IV	Intravenous
IVT/ITH	Intraventricular/Intrathecal
LPS	Lipopolysaccharide
MDR	Multidrug-Resistant
MDRO	Multidrug-resistant Organism
MIC	Minimum Inhibitory Concentration
PBP1	Penicillin-binding Protein 1
PBP3	Penicillin-binding Protein 3
PDR	Pandrug-resistant
PL	Phospholipase
SARS-CoV-2	Severe Acute Respiratory Syndrome Coronavirus-2
SSTI	Skin And Soft Tissue Infection
TDM	Therapeutic Drug Monitoring
UTI	Urinary Tract Infection
VAP	Ventilator-Associated Pneumonia
WHO	World Health Organization
XDR	Extensively Drug-Resistant
